# Dysregulation of Alternative Poly-adenylation as a Potential Player in Autism Spectrum Disorder

**DOI:** 10.3389/fnmol.2017.00279

**Published:** 2017-09-13

**Authors:** Krzysztof J. Szkop, Peter I. C. Cooke, Joanne A. Humphries, Viktoria Kalna, David S. Moss, Eugene F. Schuster, Irene Nobeli

**Affiliations:** ^1^Department of Biological Sciences, Institute of Structural and Molecular Biology, Birkbeck, University of London London, United Kingdom; ^2^The Institute of Cancer Research London, United Kingdom

**Keywords:** autism spectrum disorder, alternative poly-adenylation, RNA–seq, calcium signaling, transcription

## Abstract

We present here the hypothesis that alternative poly-adenylation (APA) is dysregulated in the brains of individuals affected by Autism Spectrum Disorder (ASD), due to disruptions in the calcium signaling networks. APA, the process of selecting different poly-adenylation sites on the same gene, yielding transcripts with different-length 3′ untranslated regions (UTRs), has been documented in different tissues, stages of development and pathologic conditions. Differential use of poly-adenylation sites has been shown to regulate the function, stability, localization and translation efficiency of target RNAs. However, the role of APA remains rather unexplored in neurodevelopmental conditions. In the human brain, where transcripts have the longest 3′ UTRs and are thus likely to be under more complex post-transcriptional regulation, erratic APA could be particularly detrimental. In the context of ASD, a condition that affects individuals in markedly different ways and whose symptoms exhibit a spectrum of severity, APA dysregulation could be amplified or dampened depending on the individual and the extent of the effect on specific genes would likely vary with genetic and environmental factors. If this hypothesis is correct, dysregulated APA events might be responsible for certain aspects of the phenotypes associated with ASD. Evidence supporting our hypothesis is derived from standard RNA-seq transcriptomic data but we suggest that future experiments should focus on techniques that probe the actual poly-adenylation site (3′ sequencing). To address issues arising from the use of post-mortem tissue and low numbers of heterogeneous samples affected by confounding factors (such as the age, gender and health of the individuals), carefully controlled *in vitro* systems will be required to model the effect of calcium signaling dysregulation in the ASD brain.

## Introduction

Transcription of eukaryotic genes ends with the recognition of a cleavage and poly-adenylation site in the genomic sequence that signals to the transcription machinery the “end” of the gene. APA, whereby multiple sites in the same gene compete for this role resulting in transcripts with different 3′ UTR lengths, is recognized as a widespread mechanism for regulating gene expression (Elkon et al., 2013). Approximately half of all human genes have more than one poly(A) site (Tian et al., 2005) and this number is likely to be an underestimate, as indicated by more recent sequencing experiments (Müller et al., 2014). Moreover, these sites are differentially selected in different tissues (MacDonald and McMahon, 2010; Lianoglou et al., 2013), stages of development (Ji et al., 2009; Shepard et al., 2011) and pathologic conditions (Sandberg et al., 2008; Rehfeld et al., 2013). For example, transcripts in the brain have been shown to have the longest 3′ UTRs among all other tissues (Miura et al., 2013), whereas cancer cells seem to harbor groups of genes with both shorter (Sandberg et al., 2008; Morris et al., 2012; Xia et al., 2014) and longer 3′ UTRs (Morris et al., 2012), each group enriched for different functional categories.

Increasing evidence of the role of APA in disease has led us to explore its potential involvement in ASD. Tentative links between ASD and APA have appeared in recent studies but all such studies have concentrated on 3′ UTR variation in *individual* genes (**Table 1**). For example, mutations in the *MECP2* gene, a transcriptional regulator, have been identified as the primary cause of Rett Syndrome, a neurological disorder that shares many of its symptoms with ASD. Although mutations in the coding region account for the majority of Rett cases, an estimated 25–35% of patients do not have such mutations, suggesting that their defects may be in non-coding regions (Newnham et al., 2010), and mutations in the 3′ UTR of *MECP2* have been linked to this disorder (Hoffbuhr et al., 2001). Of particular interest to the study of neurodevelopmental disorders such as ASD is the fact that the *MECP2* 3′ UTR is known to be developmentally regulated, with complex patterns of expression in the mouse differentiating brain (Coy et al., 1999) and age-dependent patterns in the human brain (Balmer et al., 2003; Han et al., 2013). A mechanism by which mutations in the untranslated region of this gene might lead to dysregulation of APA and consequently gene function, has been put forward by (Newnham et al., 2010) whose study showed that mutating a G-rich *cis*-acting element in the gene’s 3′ UTR strongly affects the utilization of the proximal poly-adenylation site. A more direct link between 3′ UTR mutations in the *MECP2* gene and autism was suggested by (Shibayama et al., 2004) but their study included only 24 autistic samples. The larger study by (Coutinho et al., 2007) also found variations in the 3′ UTR sequence but only in a small subset of ASD patients. Despite the strong bias toward the study of coding regions and copy number variations, especially in older studies, *MECP2* is not the only gene whose 3’ UTR has been linked to autism. Isoforms of *FMR1*, the gene associated with Fragile X Syndrome, an inherited condition characterized by autistic phenotypes, are produced in the brain from three distinct poly-adenylation sites, but relative usage of these sites is perturbed in mouse models of gene variants with increased CGG repeat lengths in their 5′ UTR (Tassone et al., 2011), suggesting a possible connection between selection of the poly-adenylation site and Fragile X-related pathologies. In summary, there is some evidence from previous studies that variations in the 3′ UTR affecting APA of *individual* genes may be linked to autistic or autism-related phenotypes. However, the hypothesis we present here is novel in two ways: (a) we suggest that dysregulated APA is a downstream effect of some other molecular disruption in ASD (and not the cause of it) and (b) we examine the possibility of an abnormal APA mechanism in the ASD brain, potentially affecting *multiple* genes and contributing to the complex pathology of this disorder.

**Table 1:** Brief summary of selected publications reporting data that support parts of our hypothesis.

	Reference	Brief summary of the link to our hypothesis
Studies linking the 3′ UTR or APA of individual genes to disorders with autistic phenotypes	Hoffbuhr et al., 2001	This study is the first report of a genomic rearrangement implicating the 3′ UTR of the *MeCP2* gene in Rett syndrome.


	Shibayama et al., 2004	The authors analyzed variants in the conserved regions of the 3′ UTR of the *MeCP2* gene in patients with autism, schizophrenia and other psychiatric diseases and found that in the case of autistic patients the frequency of mutations was higher among affected individuals than among controls, suggesting that the untranslated tail of this gene could be implicated in ASD.
	Coutinho et al., 2007	First study to scan the whole of the 3′ UTR of the *MeCP2* gene in autistic patients. The authors reported 12 novel mutations in the 3′ UTR of this gene.
	Newnham et al., 2010	The authors suggest that, since 25–35% of Rett patients do not have mutations in the coding parts of *MeCP2*, they are likely to have instead variants in other parts, including the 3′ UTR.
	Tassone et al., 2011	This study shows that the 3′ UTR of brain *FMR1* premutation alleles (linked to Fragile X-associated syndromes) is regulated differently to the UTR of normal alleles, with differential usage of poly-adenylation sites observed in the premutation tissue samples.
Studies linking ASD to aberrant calcium signaling	Splawski et al., 2004	In this study Timothy syndrome, a disorder associated with multiple dysfunctions including autism, is linked to a single missense mutation in the L-type calcium channel Ca_v_1.2. Experiments of the same group showed that this mutation impairs voltage-dependent inactivation of the channel and leads to only partial inactivation compared with the wild type.
	Krey and Dolmetsch, 2007	In this review, the authors summarize evidence that links autism to aberrant Ca^2+^ signaling during the development of the central nervous system. Several genes are discussed, including voltage-gated calcium channels, voltage-activated Na+ channels, neurotransmitter receptors and signaling proteins. The authors also point out that mutations associated with ASD tend to increase Ca^2+^ signaling, suggesting that the disorder may arise from excessive activation of networks controlled by calcium.
	Palmieri et al., 2010	This study concentrates on the gene SLC25A12, which encodes the major isoform of mitochondrial asparate/glutamate carrier (AGC) in the brain, a protein whose activity is regulated by intracellular calcium. This protein transports glutamate into mitochondria, which in turn controls the respiratory chain and production of ATP by oxidative phosphorylation. The authors show that AGC activity is increased threefold in autistic brains in the absence of a corresponding increase in protein levels. They also show that this increase is most likely due to increased Ca^2+^ levels in the autistic samples, whilst genetic variants of the SLC25A12 locus are shown not to have an effect on AGC activity in the samples studied.
	Lu et al., 2012	In this study, SNPs in ten calcium channels are tested for their association with ASD using data from over 2000 families affected by the disorder. Four SNPs in three calcium channels are found to be associated with autism.
	Schmunk et al., 2015	In this study calcium release mediated by inositol triphosphate (IP_3_) receptor channels is found to be substantially reduced in fibroblasts from autistic individuals as compared with those from controls.
Studies linking calcium signaling to APA	Flavell et al., 2008	This study focuses on an activity-dependent gene program that controls the development of synapses and is regulated by the transcription factor *MEF2*. It is shown that neuronal activity affects the choice of the polyadenylation site, favoring proximal sites and resulting in shorter isoforms for many *MEF2* target genes. One way of activating the *MEF2* gene is through calcium influx, following increased release of neurotransmitters at synapses.
	Chakrabarti and Hunt, 2015	In this study it is proposed that the plant homologue of the key poly-adenylation factor *CPSF30* in mammals is regulated by calcium in a process mediated by calmodulin binding. Calmodulin acts as a calcium sensor and its binding to *CPSF30* inhibits the RNA-binding properties of this factor, providing a link between calcium and poly-adenylation in plants.
Studies linking kinetics of Pol II to selection of poly-adenylation site	Pinto et al., 2011	In this study the effect of transcription kinetics on poly-adenylation site selection is demonstrated with the use of a *Drosophila* mutant, whose Pol II elongation rate is reduced when compared with the wild type. These mutant flies favor the proximal poly-adenylation site on the cell-cycle kinase product of the *polo* gene, as well as on another five *Drosophila* genes with tandem poly-adenylation sites in their 3′ UTRs.
	Yu and Volkert, 2013	In this study a drug that is known to decrease the rate of transcription elongation in yeast is used to demonstrate that the effect of UV damage on poly-adenylation site selection in the *RPB2* gene is most likely due to changes in the speed of elongation as opposed to changes in transcription induction.

*This table is not meant to be comprehensive but instead highlights literature that inspired us to construct the hypothesis presented here*.

## Abnormal Calcium Signaling May Lead to Dysregulation of Alternative Poly-Adenylation in the Autistic Brain

**FIGURE 1:**
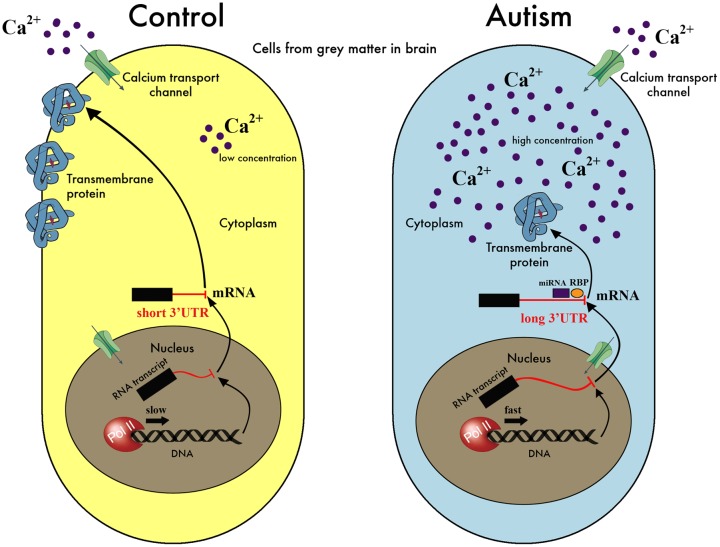
A model for the hypothesis of APA dysregulation in the autistic brain. Our hypothesis suggests that a disruption of regulation of calcium levels in the cell affects the kinetics and pausing of Pol II, resulting in aberrant selection of poly-adenylation sites during the transcription process. In this simplified schematic, an excess of calcium in the autistic cell is signaled to the nucleus increasing the speed of Pol II and leading to preferential selection of the distal over the proximal poly-adenylation sites. This, in turn, results in the inclusion of regulatory elements in the 3′ UTR, such as binding sites for miRNA or RNA-binding proteins (RBP), at times when their presence is undesirable. These elements affect the translation efficiency or degradation rate of the mRNA in question potentially leading to less protein product, or interfere with localization signals leading to misplaced protein. As we have no evidence for specific genes/proteins playing a major role in the hypothesis, we have deliberately avoided labeling the molecules in this cartoon of our model. It is also important to highlight that this is only one possible scenario that is compatible with our hypothesis but other possibilities exist, e.g., calcium levels may go down instead of up, the transcription process may lead to shorter rather than longer mRNAs, the amount of protein produced may or may not be affected etc. In fact, it is likely that several possibilities co-exist and are applicable to different genes, depending on the presence and levels of other factors, as well as the sequence signals integral to the mRNAs in question.

We present here the hypothesis that a downstream effect of the reported abnormalities in calcium signaling in ASD individuals (Krey and Dolmetsch, 2007; Lu et al., 2012; Palmieri et al., 2010; Schmunk et al., 2015, 2017) would be the dysregulation of the mechanism of APA. The role of calcium signaling in determining transcript structure and expression level in neural cells is well documented both at individual gene (Zacharias and Strehler, 1996; Poulsen et al., 2004; Rozic-Kotliroff and Zisapel, 2007) and genome-wide scales (McKee et al., 2007). In our proposed model, disruptions in the calcium signaling response lead to abnormal RNA polymerase II (Pol II) kinetics and pausing, with consequences on the selection of 3′ UTR lengths, and concomitant disruption of the regulation of mRNA localization, stability and translation (**Figure 1**). Disrupted calcium homeostasis, either through disruption of the release of intracellular stores or defects in the channels controlling the entry to cytoplasm from the extracellular space, has been suggested as one of the problems affecting autistic individuals (Krey and Dolmetsch, 2007; Palmieri et al., 2010; Schmunk et al., 2015), and mutations in a calcium channel is the single source of pathology in the Timothy Syndrome (Splawski et al., 2004), a disorder strongly associated with ASD.

The link between calcium concentration and APA site selection is most likely a consequence of the role of calcium signaling on transcriptional program activation (Ebert and Greenberg, 2013), which is known to impact on the selection of the poly(A) site (Ji et al., 2011). Distinct transcriptional programs dictate the production of different isoform ratios and consequently, the dysregulation of such programs through aberrant signaling could be expected to impact on these ratios. A number of studies support a pathway linking calcium signaling to the selection of poly-adenylation site. In cultured hippocampal neurons from rat embryos, neuronal activity via calcium signaling triggers Pol II pausing, resulting in increased selection of the proximal site in a number of genes targeted by MEF2, a family of regulators of synapse development (Flavell et al., 2008). Interestingly, genetic variants of *Mef2c* are linked to ASD and *Mef2c* mutant mice display behaviors reminiscent of this disorder (Harrington et al., 2016). Calcium has also been implicated in poly-adenylation in plants: the RNA binding activity of the plant homologue of the mammalian poly-adenylation factor *CPSF30* is regulated by calmodulin binding in a calcium-dependent manner (Chakrabarti and Hunt, 2015). Pol II kinetics also play a role in the selection of the poly(A) site: a slower Pol II mutant in *Drosophila* was associated with more efficient use of the proximal poly-adenylation site (Pinto et al., 2011) and a drug-induced reduction in transcription elongation rate alleviated the effect of preferential selection of the distal poly(A) site in the *RPB2* yeast gene following UV damage, suggesting that the rate of transcription plays a major role in APA (Yu and Volkert, 2013). In summary, there are established links between all stages of our model, from calcium signaling and neuronal activity to Pol II kinetics and the control of APA (**Table 1**). Additional regulatory mechanisms such as histone modifications and chromatin restructuring may also constitute plausible links in this chain of events (Spies et al., 2009; Huang et al., 2013; Lee and Chen, 2013) and their role in aberrant APA should be probed further in future experiments.

Although our hypothesis suggests calcium signaling abnormalities as a potential source for aberrant APA events, we do not claim that this is the only explanation compatible with previous studies or the data presented here. A great number of protein factors are involved in poly-adenylation and cleavage but we believe that changes to the expression of isolated factors are unlikely to be the culprit as these factors do not appear in ASD risk gene lists and they are not highlighted in differential expression studies of the autistic brain transcriptome. A more likely culprit, in our opinion, would be a general cell response to stress. In a recent study, Hollerer et al. (2016) showed that ribotoxic stress promoted the usage of distal poly(A) sites and suppressed proximal sites in a mammalian cell line. As this result is in agreement with previous work linking stress response to increased selection of distal poly(A) sites (Graber et al., 2013; Liu et al., 2013), Hollerer et al. (2016) concluded that APA is a widespread stress response mechanism. Although this is an appealing explanation for the presence of differential APA events in the autistic brain, it offers no molecular links between the stressed cell and altered poly(A) site usage. In this aspect, we believe that our hypothesis goes one step further in providing a plausible molecular mechanism for differential poly(A) site selection in ASD.

It is important to point out that we do not claim dysregulation of APA to be the cause of autism and have no evidence to support such a claim. Our hypothesis suggests that differential APA events are a downstream effect of some other dysregulated mechanism or possibly, a combination of such mechanisms. We suggest that these mechanisms might involve calcium signaling, as pathways controlled by calcium homeostasis are both known to be disrupted in ASD and be responsible for the regulation of transcriptional and post-transcriptional events. Our limited access to experimental data does not allow us to confirm a link between calcium and differential APA in autism, however, we argue below that such links are testable with the right experimental set ups.

## Evidence Supporting the Hypothesis of APA Dysregulation in the Autistic Brain

### Significant Differential APA Events Are Found in Transcriptomic Studies of Post-mortem ASD Brain

**FIGURE 2:**
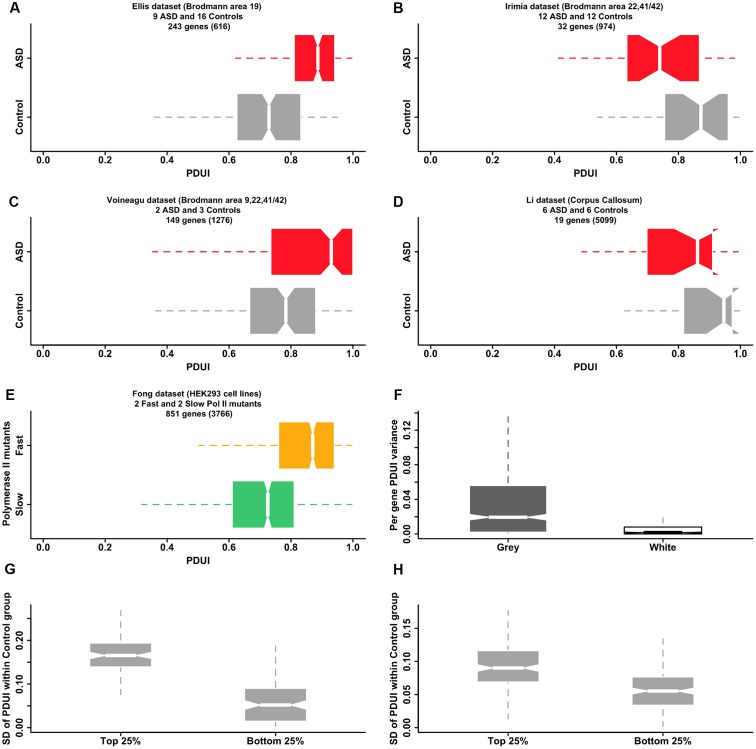
Publicly available transcriptomic data supports a model of APA dysregulation in the ASD brain. **(A–D)** RNA-seq data for ASD and control post-mortem brain samples from four publicly available datasets show statistically significant differential APA events, as revealed by the software DaPars-v.0.9 (Masamha et al., 2014). Box-and-whisker plots depict the distribution of Percentage Distal poly(A) site Usage Index (PDUI) values within a set of samples, grouped by condition (gray:control; red:ASD). The PDUI value is calculated for each gene passing the coverage thresholds of DaPars (number of genes quoted in brackets) and is a measure of the preference of using the distal over the proximal poly(A) site (PDUI is 1, if all reads are assigned to the distal site and 0, if all reads are assigned to the proximal site). Only statistically significant events (adjusted *p*-value < 0.05, Fisher exact test) are included in the box-and-whisker plots. There is a statistically significant preference for longer 3′ UTRs (larger PDUI values) in the ASD group as compared with the control group in both the *Ellis* and *Voineagu* datasets (*p*-value < 2.2e-16 and *p-value* = 6.2e-12 respectively for one-sided KS test between control and ASD group mean PDUIs). The *Irimia* and *Li* datasets show the opposite trend (*p*-values are 0.0018 and 0.075 correspondingly) but much smaller numbers of differential events have been identified in these datasets (see also **F** of this figure). **(E)** “Fast” mutants of Pol II affect the selection of the poly-adenylation site in a way reminiscent of the effects observed in ASD brain samples. Box-and-whisker plots show the distribution of PDUI values for genes showing differential APA events between “fast” (orange) and “slow” (green) mutants of Pol II (data from (Fong et al., 2014)). There is a clear preference for longer 3′ UTRs (larger PDUI values) in the case of the fast mutant (*p*-value = 2.9e-06, KS test), reminiscent of the ASD group in the *Ellis* and *Voineagu* datasets **(A,C)**. **(F)** Samples originating from brain gray matter display larger variations in PDUI values compared with samples from white matter (*p*-value < 2.2e-16; unpaired one-sided Wilcoxon rank-sum test), offering an explanation for why we find so few differential APA events in the *Li* dataset (which originates from *corpus callosum*, an area of the brain dominated by white matter). The box-and-whisker plots display the range of PDUI variances calculated by DaPars for 1038 genes from the *Mills* dataset. The dataset originates from (Mills et al., 2015) and comprises three samples derived from white matter and three samples derived from gray matter. The plot suggests that samples from white matter can be expected to show relatively little variation in their PDUI values. **(G,H)** Genes associated with greater variance in APA site selection among neurotypical subjects also show larger PDUI group mean differences between ASD and control subjects. Box-and-whiskers plots of the standard deviation of PDUI value within the control group are shown for genes with the largest difference in APA site selection between ASD and control groups (top 25% of the distribution of absolute values of PDUI group mean differences) and genes with the smallest difference (bottom 25% of the same distribution). Plots are shown for the *Ellis* **(G)** and *Irimia* **(H)** datasets but plots for the *Voineagu* and *Li* datasets are similar (not shown). Genes with large between-group PDUI differences show much larger variability in the PDUI value within the control group in both the *Ellis* and *Irimia* datasets (*p*-value = < 2.2e-16 for both datasets; unpaired, one-sided Wilcoxon rank-sum test). All plots were generated using the R statistical software suite. The boxplot whiskers extend in all cases to the most extreme data point, which lies at no more than 1.5 times the interquartile range from the box.

Our hypothesis raises the question of whether it is possible to find direct evidence of APA dysregulation in the autistic brain in publicly available transcriptomic datasets. Microarray-derived datasets are currently not amenable to this type of analysis due to both lack of probes covering all areas of potential interest and the great variability in individual probe signal that is practically impossible to normalize effectively. Our analysis of four RNA-seq datasets from the literature (**Figures 2A–D**) supports our hypothesis for the case of gray matter originating from selected regions of the brain but the diversity of the signal reflects partly the difficulty in using data that was not designed for the specific probing of APA. Further analysis of RNA-seq data from the Fong et al. (2014) study adds support to our suggested mechanism of APA dysregulation: changes to the elongation rate of Pol II affect the selection of the poly-adenylation site. Pol II slow and fast mutants show opposite trends in the 3′ UTR length of their transcripts, when compared with the wild type polymerase, with the fast mutant lengthening the 3′ UTR in a manner that resembles the effect observed in two of the ASD/control datasets (**Figure 2E**). Although the origin of the changes in the Pol II rate are different in the Fong et al. (2014) study (mutations in the polymerase gene) and in our hypothesis (dysregulation of mechanisms such as calcium signaling), the end result is the same: detectable changes to APA. Hence, both the literature and existing transcriptomic data provide evidence supporting our model that links dysregulation of calcium homeostasis in the ASD brain with poly(A) site switching anomalies, mediated by calcium-dependent signaling pathways controlling the speed and pausing of Pol II.

An additional question raised by our hypothesis is what are the molecular factors mediating aberrant APA site selection, given that only a subset of genes are affected and the size of the effect varies between genes? Our search for sequence motifs, fold stability and distance between sites have all resulted in only weak correlations with APA selection dysregulation. There is some evidence that expression of some poly-adenylation factors is linked to the APA anomalies but no single gene stands out. The most interesting link we have found is that between variability in APA site selection among samples from healthy individuals and the size of the differences in site selection between ASD and control samples (**Figures 2G,H**). Based on this observation, we suggest that there is a subset of genes that are more susceptible to environmental or genetic factors affecting poly-adenylation site selection (possibly due to less tight regulation), and that it is this set of genes that exhibit the largest APA anomalies in the case of disease. In our model, normally harmless variations become amplified or combine with other aberrant events potentially contributing to pathological conditions.

### Genes Exhibiting Differential APA Events Are Enriched for Calcium-Driven Signal Transduction, the Immune System and the Cytoskeleton

The observation of differential APA events in the autistic brain led us to explore further the genes involved in an effort to identify potential biases in the pathways affected by these events. We searched first for overlap of our gene lists (of significantly differential APA events in either the *Ellis* or *Voineagu* datasets) with the 719 ASD candidate genes listed in the SFARI Gene module (Abrahams et al., 2013). Interestingly, no significant overlap was found (*p*-value > 0.1, hypergeometric test), suggesting that APA dysregulation is distinct from genetic factors known to be associated with ASD. Instead, our two lists are significantly enriched in both synaptic (*p*-value < 1.0e-11 for *Ellis*, <1.0e-3 for *Voineagu*) and post-synaptic density proteins (*p*-value < 1.0e-24 for *Ellis*, <1.0e-11 for *Voineagu*) (Bayés et al., 2011) and show, in addition, significant overlap (*p*-value < 1.0e-8 for *Ellis*, <1.0e-5 for *Voineagu*) with neuronal genes responding to intracellular Ca2+ concentration (McKee et al., 2007). Gene names for significantly overlapping lists are given in Supplementary Table 1.

**FIGURE 3:**
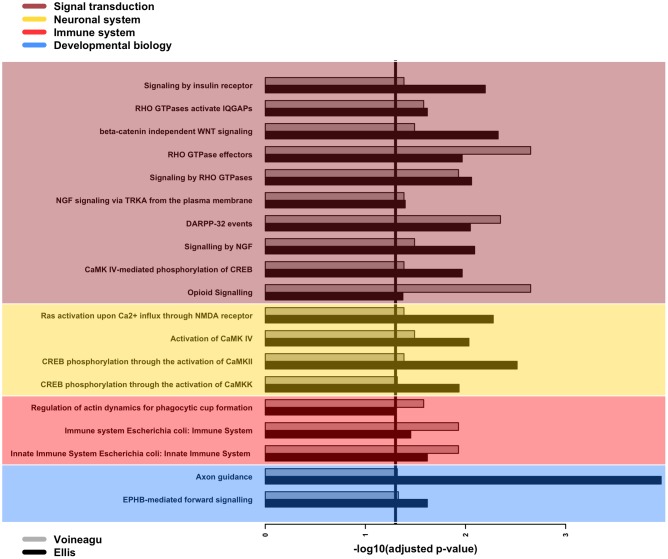
GOseq enrichment of Reactome pathways among genes with differential APA events. Barplot of adjusted *p*-values for the enrichment of 19 Reactome pathways over-represented among genes with differential APA events in both *Ellis* and *Voineagu* datasets. The pathways have been grouped according to their main classification within Reactome, with signal transduction being the most commonly occurring classification. The vertical line indicates the chosen significance cut-off (adjusted *p*-value < 0.05).

Next, we searched for over-represented pathways and gene ontology (GO) terms in our list of affected genes using the Bioconductor package GOseq (Young et al., 2010). We examined 29 genes associated with statistically significant APA events in both the *Voineagu* and *Ellis* datasets (Supplementary Table 2). The set of all expressed genes in each dataset was used as the background set in each calculation. The analysis pointed to 19 Reactome pathways (**Figure 3**) that are enriched in both datasets, with a strong bias toward signal transduction. More specifically, several of these pathways relate to signaling events mediated by Rho-GTPases as well as the calcium-binding calmodulin genes (*CALM1/2/3*) and genes controlled by the cyclic AMP-response element (CRE)-binding protein (CREB). Further support for the involvement of CREB pathways comes from the fact that our lists of genes affected by APA dysregulation are enriched in the presence of the CRE element in their promoters (*p*-value = 0.0001 and 0.006 for the *Ellis* and *Voineagu* datasets respectively; hypergeometric test). Moreover, there are enriched pathways that are linked to important aspects of brain development, such as axon guidance (Reactome ID: R-HSA-422475) and dendritic spine development (R-HSA-3928662). The innate immune system is also directly implicated in this list (R-HSA-168249, R-HSA-2029482), as is the actin cytoskeleton (R-HSA-2029482). It is worth noting that these over-represented pathways involve only 14 of the genes in the list of 29 (*CALM3, CALM1, PPP2R1A, RHOA, ACTB, ARPC1A, EVL, CTSF, HLA-A, PEBP1, CAMK2B, GNB2, ATP6V0C, NGFRAP1*). The remaining genes either do not map to Reactome pathways or map to pathways that do not pass the enrichment filters.

Interestingly, the pathways identified as enriched in APA dysregulation events recapitulate the findings of several recent studies that highlight molecular interaction networks affected by genes implicated in ASD. For example, cyclic AMP signaling is one of the two central hubs in a network of molecular interactions revealed by analysis of gene expression perturbations resulting from knockdown of a number of ASD-associated genes in mouse primary neuron cultures (Lanz et al., 2013). The cytoskeleton features as one of the three major clusters in the analysis of biological pathways perturbed in ASD as a result of *de novo* mutations (Chang et al., 2015) and several GO terms relating to actin organization are associated with a functional network revealed in a separate study of rare *de novo* copy number variations (CNVs) in ASD patients (Gilman et al., 2011). Moreover, the stability of microtubules, a major constituent of the cytoskeleton, is impaired in cell cultures from Rett patients affected by MeCP2 deficiency (Delépine et al., 2013). It is also worth pointing out that signaling by Rho GTPases (one of our enriched Reactome pathways) is linked to actin network remodeling (Saneyoshi and Hayashi, 2012), and hence this pathway too converges to a network relating to the cytoskeleton.

To what extent the overlap between ASD-linked pathways and APA dysregulated ones is coincidental is difficult to establish statistically, not least because the true range of networks affected by ASD is not known. Hence, the examples highlighted above are not employed here to lend extra support to our hypothesis. However, if the hypothesis is tested and turns out to be true, links to networks could be useful in focusing further tests, helping to predict downstream effects or even helping to rationalize selected phenotypic observations in ASD by linking them to molecular-level abnormalities.

### Gene Set Enrichment Analysis Links APA Dysregulation to Pathways with Established Links to ASD

We carried out gene-set enrichment analysis (GSEA) replacing the more commonly used gene expression values in this type of analysis with the PDUI values calculated by DaPars (a PDUI value essentially measures the preponderance of the long isoform over the short isoform in a two-site APA model). We found no enrichment of the MSigDB (Subramanian et al., 2005) “hallmark gene sets” using the *Voineagu* dataset, possibly due to the very low number of samples in each phenotype group. However, in the *Ellis* dataset, four of 16 gene sets that pass the size filters from the hallmark sets are significantly enriched (FDR *q*-value < 25%) among the ASD samples: adipogenesis, oxidative phosphorylation, fatty acid metabolism and apoptosis. Links of ASD to adipogenesis and fatty acid metabolism are well-established in the literature (Tamiji and Crawford, 2010), with a syndromic form of the disorder (Prader–Willi) being strongly affected by obesity, a problem that is also manifested in mouse models of ASD (Liu et al., 2015). Apoptosis has also been linked to autism and several relevant studies are summarized in the review of (Wei et al., 2014). Finally, an enrichment of the oxidative phosphorylation gene set is also not unexpected as some studies point to a link between mitochondrial disease and autism (e.g., Weissman et al., 2008; Giulivi et al., 2010). Hence, all the enriched groups have known associations with ASD. No significant enrichment is observed for the control samples.

## Suggestions for Further Testing of the Hypothesis

Given our findings, extending the study to any other transcriptomic datasets from autistic brain seems like an obvious next step. However, our pilot studies of publicly available RNA-seq datasets have revealed several caveats of using RNA-seq to study APA. First, software that identifies APA sites from the distribution of reads mapping to the 3′ UTR requires a good coverage of that region. Hence, current methods are likely to discover more events in libraries resulting from poly(A) selection of RNA rather than the recently more popular ribosomal RNA depletion kits, as the latter exhibit a bias against the 3′ end which weakens the signal from APA events. Second, the samples must be prepared from areas of the brain with predominantly gray matter, as trends appear much weaker in a brain dataset consisting primarily of white matter (**Figure 2F**). However, which regions of the brain would be most suitable for observing larger effects remains unknown. Third, the samples of a dataset should be as closely matched as possible, because confounding variables such as age and gender add unwanted variability in the signal, likely concealing a small in magnitude but potentially widespread across many genes signal. Moreover, differential APA events may only be detectable at certain stages of brain development but most datasets of post-mortem tissue inevitably comprise a range of ages, potentially diluting the signal that could be detected in a subset of these samples. Finally, post-mortem samples are plagued by low quality issues due to degradation, and such problems severely hinder the downstream analysis of sequencing data.

Given the difficulty in predicting APA events from standard RNA-seq data, we suggest that the initial test of this hypothesis should focus on confirming dysregulation of the APA mechanism using a method that measures a signal from the actual poly-adenylation site. The majority of these methods work by capturing the poly-adenylation tail of transcript fragments, followed by sequencing a short segment of the nucleotides directly attached to it. An advantage of these methods is that they can discover novel events, including those that involve lengthening of the 3′ UTR beyond the longest known annotated isoform (which is often used as the “reference” transcript in computational methods searching for read density fluctuations in RNA-seq data). Methods like PAS-seq (Shepard et al., 2011), 3′-seq (Jenal et al., 2012) or Poly(A)-seq (Derti et al., 2012) (and others summarized in the review by Sun et al., 2012) would be reasonable choices, although the protocols require special expertise and these methods are not usually offered by services. The introduction of kits that allow easy library preparation (such as Lexogen’s QuantSeq) is a promising step toward making 3′ sequencing easily accessible to more labs. Direct RNA sequencing (DRS) (Ozsolak et al., 2009), a single-molecule sequencing approach that provides reads starting from the poly-adenylation site is also appealing because it requires much smaller amounts of RNA and avoids the PCR amplification step, which introduces biases in the quantification of expression. DRS experiments have been used to quantify differences in the use of alternative sites (Ozsolak et al., 2010; Schurch et al., 2014) and are thus suitable for testing our hypothesis. The more recent PAPERCLIP (Hwang et al., 2016) method may be also an attractive alternative, largely due to its potential for cell-type-specific 3′end mapping. A caveat of using either RNA-seq or targeted 3′ sequencing data is that they are both blind to post-transcriptional processing of RNA that generates transcripts from the 3′ UTR, expressed independently of the protein-coding part of the gene. The existence of such transcripts has been suggested, backed up by both computational and experimental evidence (Mercer et al., 2011). Methods that detect and quantify the expression of full-length transcripts using ultra-long reads (e.g., the technologies available from Pacific Biosciences or Oxford Nanopore) would allow the detection of these 3′ UTR RNAs and their consideration as separate species to the traditional transcripts produced by the same gene locus.

It is important to point out that, for all the reasons stated at the beginning of this section, isolating APA events identified in RNA-seq data and testing for their presence in any but the samples in which they have been actually observed is likely to be a pointless exercise. However, if dysregulated events were found using a more direct method such as DRS, then isolating genes with large effect sizes and focusing on validating alternative isoforms with qRT-PCR would be advisable.

The data presented in this study come from brain samples. Post-mortem brain tissue is a valuable resource, not trivial to access and of limited availability. It could be argued that blood-derived samples would be easier to obtain but there is no evidence they would recapitulate effects stemming from dysregulation of signaling pathways in the brain. Moreover, there is evidence that a limited number of transcripts are detectable in blood (GTEx Consortium, 2015) making this medium a less appealing choice. A number of mouse models carrying mutations or deletions affecting ASD-linked genes are also available (Provenzano et al., 2012). Although some researchers have debated the adequacy of the mouse brain as a suitable model for complex diseases (Miller et al., 2010), rodent models have been used successfully in several studies of autism and related pathologies, including the exploration of behavioral and physiological changes as well as the testing of pharmacological interventions in *Fmr1* (Gandhi et al., 2014), *Cntnap2* (Peñagarikano et al., 2011) *and Tsc2* knock-out mice (Ehninger et al., 2008) (a comprehensive review of such studies is given in (Crawley, 2012)). A viable alternative to post-mortem tissue and animal models may be the use of induced pluripotent stem (iPS) cells (Ardhanareeswaran et al., 2017; Kathuria et al., 2017) or even mini-brains that are already being used to probe serious neurological conditions (Kelava and Lancaster, 2016) and are expected to become much more accessible as model systems in the near future. We believe the absence of confounding factors affecting the brain samples such as medication, degradation of the tissue, and age of the donors would make *in vitro* set ups a good platform on which to study APA in the context of autism. Additionally, studies of this kind allow for the use of small molecules that can modulate signaling pathways (e.g., interfering with the calcium signaling processes), thus making it easier to design experiments of the effect of calcium dysregulation on APA.

## Possible Implications of APA Dysregulation in the Autistic Brain

If a dysregulated signaling network affected the selection of poly-adenylation site in the autistic brain, what would be the implications of such dysregulation? One would expect changes in the length of the 3′ UTR to have some effect on gene expression, primarily through miRNA binding which might be especially important in the brain, as this is the tissue with the longest 3′ UTR and hence greatest potential for miRNA regulation. As miRNA binding sites are commonly found between APA sites (Sandberg et al., 2008; Hoffman et al., 2016), an extension of the 3′ UTR would be expected to lead to the inclusion of more miRNA binding sites in the transcripts, whereas shortening of the 3′ UTR would have the opposite effect. Indeed, a search of the elongated parts of the 29 UTRs affected by APA in both the *Ellis* and *Voineagu* datasets reveals at least one match to a miRNA binding site in 13 of them (Supplementary Table 3). Because miRNAs act mostly as repressors of gene expression, one could speculate that the consequence of skipping the proximal site would be less protein product for the affected transcripts. However, it cannot be excluded that homeostatic mechanisms of gene expression regulation would act to balance out the post-transcriptional effects of abnormal poly-adenylation site selection and there is evidence that changes in the isoform ratio due to APA are not correlated to changes in gene expression (Lianoglou et al., 2013). Moreover, miRNAs are not the only post-transcriptional modulators. Additional searches of the 29 elongated parts reveal the presence of a variety of UTR elements in 16 of the genes and a large number of motifs that could be targets of RNA-binding proteins in each of the 29 UTR extensions (Supplementary Table 3). Although it is difficult to assign any statistical significance to the presence of these motifs, we note that many of the proteins associated with these motifs are regulators of transcript processing, most prominently, alternative splicing. The 3′ UTR length is also known to affect the rate of mRNA decay although the relationship between the two is not simple, as a recent study in yeast has revealed (Gupta I. et al., 2014). Hence, not only the downstream effect on the protein level, but also the effect of APA dysregulation on mRNA stability could be debatable. An additional role of the potential influence of APA (and by extension, its dysregulation), on post-translational protein localization, independently of mRNA localization, has been demonstrated in human cell lines (Berkovits and Mayr, 2015). The suggested mechanism involves the recruitment to the site of translation of protein factors that interact with the 3′ UTR; these proteins then facilitate the translocation of the newly translated protein to its final destination. Disruption of this mechanism leads to altered spatial distribution of the relevant protein products. In neurons, where the morphology of the cell is complex, an improper balance in spatial distribution can be expected to be particularly detrimental.

We believe that the effect of APA dysregulation in the brain will depend on (a) the extent of the disruption, (b) the genetically determined ability of the individual to respond to and correct such disruptions and (c) the interplay with other factors, including medical conditions, environmental inputs etc. The effect of environmental factors is a question of particular interest, given existing studies linking environmental signals and APA in plants (Shen et al., 2011). Potentially, more relevant to the human brain is a study demonstrating that the estrogen 17-beta estradiol (E2) induces APA and shortens the 3′ UTR of *CDC6*, resulting in higher levels of the protein product that regulates DNA replication (Akman et al., 2012). This study raises the question of whether APA regulation may differ between males and females, where E2 levels are higher, and whether such differences may also play a role in the relationship between APA and autism, given that this disorder has a strong prevalence among males. The link between APA and estrogens in general could also be worth investigating further in the context of the interplay between genetics and environment. Given the increasing levels of steroid estrogens in soil and water due to industrialization (Adeel et al., 2017), the role of estrogen pollutants and their potential link to aberrant APA in the brain may be worth investigating.

Our analysis points to a number of small changes rather than isolated APA events of significant magnitude, although we reiterate here the caveats of relying on software rather than direct experimental observation of the APA events. Small changes are unlikely to lead to observable effects, except in cases where the genetic make-up of the individual or a coexistent factor affects the cell’s ability to cope with such disruptions. However, it is possible for a large number of small changes to lead to some observable effect, and this effect can occasionally be bigger than the effect of a large change in a single gene (Mootha et al., 2003). Importantly, it has been shown that inherited common genetic variations act additively to increase the risk of ASD liability, so the possibility of polymorphisms acting to amplify a small effect in the context of this disorder is not far-fetched (Klei et al., 2012).

If our hypothesis is true, it may have implications for the early detection of ASD. It would be worth, for example, exploring 3′ UTR differences in other tissues to assess the potential for building a classifier capable of good discrimination between children affected by ASD and neurotypical children. Similarly, exploring 3′ UTR differences in other brain disorders could shed light on the question of how common APA dysregulation is among these diseases, and what, if any, are unique poly-adenylation signatures for each condition. The recent study by (Hollerer et al., 2016) suggesting increased usage of the distal poly(A) sites in cell lines as a general response to stress highlights the importance of exploring APA dysregulation in other disorders.

## Conclusion

We present here the hypothesis that the mechanism of APA is dysregulated in the autistic brain, potentially contributing to ASD pathology. We argue that the effect of dysregulated APA selection will be determined by an individual’s genetic variations and exposure to environmental factors, leading to a spectrum of effects and phenotypes, many of which would be too hard to predict without a solid understanding of the underlying molecular networks. We hope our observations and suggestions will stimulate further experiments in the field.

## Methods

### Origin of RNA-seq Datasets

The data analyzed in this project originates from six publicly available RNA–seq datasets (described in more detail below). Samples that did not pass our pre- and post-mapping data quality control (described below and in the next section) were removed. In addition, we attempted to use “cleaner” datasets by removing samples from individuals with questionable phenotypes or samples that severely biased the distribution of ages in either the condition or the control groups. One dataset (“*Ellis*”) had a very large number of samples but different areas of the brain were sampled and in some cases samples originated from the same individual. In this case, we kept only one sample per individual, to avoid biasing the group, and used only samples from the same area of the brain to minimize within-group biological differences. Briefly, our exclusion criteria were: (a) samples were removed, if they failed the quality control for sequencing bias [mean TIN value (a measure of transcript integrity equivalent approximately to RIN value × 10) across all transcripts < 40], (b) samples originating from teenager individuals in the *Ellis* dataset were removed as they were all in the control group with no corresponding-age samples in the autistic group, (c) all samples originating from any area except BA19 were removed from the *Ellis* dataset (because we did not want to have to factor in changes due to different regions of the brain), (d) all samples discarded in the original study of (Ellis et al., 2013) or in the subsequent study of the same group of authors using the same data (Gupta S. et al., 2014) were discarded here too, and (e) a handful of isolated samples for which information was available that showed the use of medication for disorders comorbid with autism were removed (to avoid including undiagnosed ASD cases as controls as well as ASD cases that would have their transcriptomes potentially severely affected by the use of drugs). Four of the datasets contained post-mortem brain samples from both healthy and ASD individuals. The *Voineagu* dataset comprises 2 autistic and 3 control samples from Brodmann areas 9, 22 and 41 (cerebral cortex) (Voineagu et al., 2011). The *Ellis* dataset contains 9 autistic and 16 control post-mortem brain samples, all from Brodmann area 19 of the occipital lobe cortex (Ellis et al., 2013). The *Li* dataset includes 6 autistic and 6 control samples from the *corpus callosum*, an area of the brain that consists predominantly of white matter (Li et al., 2014). The *Irimia* dataset comprises brain samples from Brodmann areas 41, 42 and 22 (each sample comprising a mixture of all three areas) from 12 autistic and 12 healthy individuals (Irimia et al., 2014). In addition to the above datasets, we used the *Mills* dataset of brain samples from 3 healthy individuals (Mills et al., 2015) for a comparison of APA differences between gray and white matter. Finally, we used the *Fong* dataset (Fong et al., 2014) to examine APA in isogenic human HEK293 cell lines representing RNA polymerase II mutants with accelerated and slowed down kinetics.

### Data Quality Control, Pre-processing and Alignment

The raw reads from all datasets were checked for quality using FastQC-v0.11.3 (FastQC, **RRID**:SCR_014583). Parts of the reads with low quality (defined as an average quality per base dropping below 15) and all synthetic parts of sequences, such as adaptors, that were unintentionally included during the sequencing process were trimmed using Trimmomatic v0.32 (Bolger et al., 2014). All the reads were uniquely mapped to the reference human genome (UCSC hg19 version) using Tophat2-v2.0.14 (Kim et al., 2013). Reads containing parts of the poly(A)-tail cannot be mapped to the reference genome during this first mapping round. Hence, stretches of at least three adenosines were trimmed from the end of these reads using FQtrim v.0.94 (FQtrim, doi: 10.5281/zenodo.20552) and the processed reads were mapped to the genome once again. PCR duplicates were removed with Picard-tools-v1.114 (Picard, **RRID**:SCR_006525). Finally, the output files were checked with RSeQC-v2.6.2 (Wang et al., 2012), particularly with the aim of looking for potential 3′ or 5′ bias that might indicate high RNA degradation level. In addition, TIN values for each sample were calculated as described in RSeQC. Samples with mean TIN values below 40 (after removal of zeros) were removed from the datasets. Moreover, individual transcripts were removed, if the TIN value for a particular transcript was below 60 in at least one sample.

### Assessing Differential Poly-adenylation with DaPars

Prepared files containing mapped reads were converted to *bedgraph* format using Bedtools v2.17.0 (Quinlan and Hall, 2010) and these were used as input for the software DaPars-v.0.9 (Masamha et al., 2014; Xia et al., 2014). DaPars discovers statistically significant alternative poly-adenylation events between two groups of samples (e.g., ASD vs. healthy, or white vs. gray matter etc). It predicts proximal sites using the drop in the number of reads near the site as a signal in a two-point model. In order to verify the validity of the DaPars-predicted proximal sites, a custom-made computational pipeline was applied that identifies high-confidence poly(A) sites by searching for the presence of reads with poly(A) tails terminating near the predicted site.

### Meta-Analysis of Gene Lists

Gene ontology analysis and Reactome pathway enrichment were carried out using GOseq (Young et al., 2010) to account for selection bias between long and short genes in RNA-seq. Where a background set of genes was required for enrichment analysis, we defined a subset of genes with detectable expression in each dataset. To define this subset, we estimated the expression of each gene calculating transcripts per million (TPM) values from raw counts, and considered expressed only those with median TPM > 1.

Gene set enrichment analysis (Subramanian et al., 2005) was carried out using the server available at the Broad Institute^1^ and the “hallmark gene sets” from the Molecular Signatures Database (v. 5.1). Instead of expression values, PDUI values of genes were used as input. The standard KS statistic was applied to all gene sets having at least 10 tested genes. All other settings were set to default values. The software Dreme (Bailey, 2011) from the MEME suite (Bailey et al., 2009) was used to look for over-represented motifs in the following regions: (a) 50 nucleotides upstream of the predicted proximal and annotated distal sites, (b) the region between the proximal and distal sites and (c) the region between the start of the last exon and the proximal site. In addition, motifs were searched 20000 nucleotides upstream of annotated transcription start sites and 20000 nucleotides downstream of annotated distal sites.

The program *fuzznucc* from the EMBOSS tools (Rice et al., 2000) (version 6.3.1, available from the Mobyle Pasteur server (Néron et al., 2009) was used to search for the presence of the CRE motif (TGACGTCA) or its complement in the upstream regions (20000 nucleotides upstream of the transcription start site) of (a) the genes with significant APA events (adjusted *p*-values < 0.05 from DaPars and PDUI group difference > 0.2) and (b) all expressed genes in the *Ellis* and *Voineagu* datasets.

The sequence between proximal and distal poly-adenylation site in the 3′ UTR of the 29 genes with statistically significant differential APA events in both the *Ellis* and *Voineagu* datasets was used in a series of searches for regulatory elements. The RBPmap (Paz et al., 2014) web server^2^ was used to find RNA-binding protein target motifs. The UTRScan (Grillo et al., 2010) tool^3^ was used to find UTR elements. Finally, miRNA binding sites were retrieved from the results of TargetScan (Lewis et al., 2005) available from the UCSC genome browser (Kent et al., 2002).

Lists of synaptic and post-synaptic density proteins from (Bayés et al., 2011) were obtained from the Arking group website^4^. The list of calcium–responsive genes was obtained from (McKee et al., 2007). We kept only genes with adjusted *p*-value < 0.005 (cut-off suggested in the original paper) and converted the original RefSeq IDs to gene names using the gene ID converter of the DAVID online server (Huang da et al., 2009).

Significance tests were carried out and plots were created using the R statistical software suite (R Core Team, 2015). Adjustment of *p*-values for multiple testing was carried out throughout using the Benjamini–Hochberg procedure, as implemented in R.

## Author Contributions

KS, ES, and IN conceived the idea of the project. KS and IN carried out the majority of the analysis and literature review supported by ES, DM, PC, JH, and VK. IN, ES, and DM supervised the project. IN wrote the first draft of the manuscript supported by KS, ES and DM. All authors have read and approved the final version of the manuscript.

## Conflict of Interest Statement

The authors declare that the research was conducted in the absence of any commercial or financial relationships that could be construed as a potential conflict of interest.

## Footnotes

1 http://www.broadinstitute.org/gsea/index.jsp

2 http://rbpmap.technion.ac.il/

3 http://itbtools.ba.itb.cnr.it/utrscan

4 http://www.arkinglab.org/resources/

## Abbreviations

ASD: autism spectrum disorder
APA: alternative poly-adenylation
DRS: direct RNA sequencing
KS: Kolmogorov–Smirnov (test)
PDUI: Percentage Distal poly(A) site Usage Index
Pol II: RNA polymerase II
RNA-seq: RNA sequencing.
